# Complete Genome Sequence of the Porcine Epidemic Diarrhea Virus Strain SLO/JH-11/2015

**DOI:** 10.1128/genomeA.01725-15

**Published:** 2016-04-07

**Authors:** Ivan Toplak, Manica Ipavec, Urška Kuhar, Darja Kušar, Bojan Papić, Simon Koren, Nataša Toplak

**Affiliations:** a Veterinary Faculty, Institute of Microbiology and Parasitology, University of Ljubljana, Ljubljana, Slovenia; b Omega d.o.o., Ljubljana, Slovenia

## Abstract

Porcine epidemic diarrhea virus (PEDV) was detected for the first time in Slovenia in January 2015. The complete genome sequence of PEDV strain SLO/JH-11/2015, obtained from a fecal sample of a fattening pig with diarrhea in September 2015, is closely related to recently detected European strains.

## GENOME ANNOUNCEMENT

Porcine epidemic diarrhea virus (PEDV) was first described in England in 1971 and, until the end of the 1990s, sporadic cases have been reported throughout Europe (1, 2). Since 2010, severe outbreaks with high mortality have also been reported from Asia (3). In the United States, the virus was first detected in spring 2013 and emerged through swine herds (4–7). In January 2014, a novel PEDV variant named S INDEL strain OH851 was identified in the United States (7). Between 2014 and 2015, the S INDEL strain was also identified in Germany, France, Belgium, and Japan (8–11). In December 2014, a clinical outbreak of diarrhea was observed at a pig-fattening farm, and PEDV was confirmed for the first time in Slovenia in January 2015 by real-time PCR (12). At the same farm, PEDV was detected again in September 2015, in three fecal samples collected from fattening pigs with acute diarrhea.

Direct total RNA extraction was performed using the QIAamp viral RNA kit (Qiagen, Hilden, Germany) according to the manufacturer’s instructions and subsequently using the standard TRIzol protocol. For sequencing the complete PEDV genome, an RNA library was prepared using the Ion total RNA sequencing kit version 2 (Thermo, Fisher Scientific–Ion Torrent, Carlsbad, CA, USA) according to the manufacturer’s protocol. Emulsion PCR and enrichment were carried out using the Ion PGM template OT2 200 kit (Thermo, Fisher Scientific–Ion Torrent). The amplified library was sequenced on the Ion PGM platform using the Ion PGM HiQ sequencing kit and the Ion 314 Chip version 2 (Thermo, Fisher Scientific–Ion Torrent). Reads were mapped against the reference German PEDV strain L00721 (LM645057) genome and analyzed using the DNAStar Lasergene version 10.1.1 (DNAStar, Inc., Madison, WI, USA).

The size of the PEDV strain SLO/JH-11/2015 genome was 28,028 nucleotides (nt), excluding the poly-A tail. The genomic organization was found to be the same as previously described for the isolate 15V010 from Belgium (9). The complete genome sequence of the SLO/JH-11/2015 strain was closely related to the (i) recently detected European strain GER/L00719/2014 identified in May 2014 in Germany with 49-nt changes scattering over the genome (8); (ii) strain FR/001/2014 identified in December 2014 in France with 44-nt changes (10); (iii) strain BEL/15V010/2015 with 93-nt changes; and (iv) the prototype United States INDEL strain OH851 with 185-nt changes. Our findings indicate that the PEDV strain identified in the infected herd in September 2015 is a result of circulation of the S INDEL strain previously detected in Europe.

To our knowledge, this is the first published complete PEDV sequence from Slovenia. The data confirm that the whole-PEDV-genome sequencing is possible from complex samples without the use of virus-specific primer sets. The obtained SLO/JH-11/2015 sequence will further our understanding of the genetic diversity of PEDV strains in Slovenia and facilitate future research on the epidemiology of PEDV circulating in Europe.

### Nucleotide sequence accession number.

The complete genome sequence of PEDV SLO/JH-11/2015 has been deposited to GenBank under accession number KU297956.
